# Extended Rate Constants Distribution (RCD) Model for Sorption in Heterogeneous Systems: 2. Importance of Diffusion Limitations for Sorption Kinetics on Cryogels in Batch

**DOI:** 10.3390/gels6020015

**Published:** 2020-05-14

**Authors:** Irina Malakhova, Alexey Golikov, Yuliya Azarova, Svetlana Bratskaya

**Affiliations:** Institute of Chemistry, Far Eastern Branch of Russian Academy of Sciences, 159, prosp.100-letiya Vladivostoka, 690022 Vladivostok, Russia; newira94@gmail.com (I.M.); glk@ich.dvo.ru (A.G.)

**Keywords:** polyethyleneimine, cryogel, sorption, sorption kinetic models, intraparticle diffusion, sorption dynamics, metal ions

## Abstract

Here we address the problem of what we can expect from investigations of sorption kinetics on cryogel beads in batch. Does macroporosity of beads indeed help eliminate diffusion limitations under static sorption conditions? Are sorption rate constants calculated using phenomenological kinetic models helpful for predicting sorption properties under dynamic conditions? Applying the rate constants distribution (RCD) model to kinetic curves of Cu(II) ions sorption on polyethyleneimine (PEI) cryogel and gel beads and fines, we have shown that diffusion limitations in highly swollen beads are very important and result in at least ten-fold underestimation of the sorption rate constants. To account for intraparticle diffusion, we have developed the RCD-diffusion model, which yields “intrinsic” kinetic parameters for the sorbents, even if diffusion limitations were important in kinetic experiments. We have shown that introduction of a new variable—characteristic diffusion time—to the RCD model significantly improved the reliability of sorption kinetic parameters and allowed prediction of the minimal residence time in column required for efficient uptake of the adsorbate under dynamic conditions. The minimal residence time determined from kinetic curves simulated using the RCD-diffusion model was in good agreement with experimental data on breakthrough curves of Cu(II) ion sorption on monolith PEI cryogel at different flow rates.

## 1. Introduction

Cryogels are the macroporous polymeric gels formed in moderately frozen media via polymerization/polycondensation of the monomers or covalent or non-covalent cross-linking of the polymers [1]. A broad variety of monomeric and polymeric precursors used from 1970s has allowed fabrication of cryogels for versatile applications in biotechnology, environmental science, catalysis, biomedicine, and other fields [2,3,4,5,6]. Many applications benefit, first of all, from the structure of these 3D materials with interconnected channels of large pores (with the size of tens to hundreds micrometers) ensuring superior transport properties and the possibility to separate large molecules and cells [7], which cannot be separated using macroporous resins. Most examples, when cryogels were efficient sorbents under dynamic conditions at high flow rates, were related to the recovery of highly valuable bioactive substances (proteins, enzymes, etc.), when very low binding capacity was compensated by high recovery and elution efficiency [8,9]. Recently, cryogels have been considered to be an alternative for commercially available sorbents for removal of metal ions and other pollutants from water [2,5,10,11,12]. However, in water treatment applications, advantages of the cryogel morphology have become valuable only when materials have high sorption capacities and/or selectivity and can be applied under dynamic conditions in conventional sorption set-ups at high flow rates.

Cryogels can be obtained either as beads (cryobeads) or as monoliths. Although we agree that the term “monolith” is not fully correct for cryogels, whose shape significantly depends on the compression strength and water content [1], the never-dried cryogels obtained via cross-linking or polymerization/polycondensation directly in the sorption column can be considered to be monoliths in terms of their functional properties under dynamic conditions as long as liquid flow does not induce morphological changes. There are no principal limitations to use cryobeads in sorption column applications, but cryogels are usually soft, highly deformable materials lacking mechanical stability under stirring or high flow rates. In most studies on cryobeads, this aspect was avoided, and sorption properties were reported only for static conditions [12,13,14,15], while mechanical properties of the beads and sorption dynamics were not investigated. Önnby et al. have shown that despite very good sorption properties of IDA-modified cryogels toward transition metal ions, they cannot be used as obtained and must be fabricated in specially designed plastic carriers to have sufficient mechanical resistance under stirring [16]. The same approach was used for the application of composite cryogels for bromate recovery [17]. Surprisingly, examples of cryogel performance in sorption under dynamic conditions are very limited, with breakthrough curves often obtained only at one flow rate [18,19,20] or from very diluted solutions without reaching saturation [21]. Despite the assumption that sorption on cryogels is free from diffusion limitations [2,22], a significant reduction of the dynamic adsorption capacities with an increasing flow rate was observed for chelating cryogels [23,24]. The sorption rate of Pb(II) ions on TiO_2_ particles was notably lower, when nanoparticles were embedded into the cryogel [19]. The attempt to identify the limiting stage of sorption showed in this case several linearity regions and significant influence of intraparticle diffusion.

Aside from the external and intraparticle diffusion limitations, the reaction rate can be also a limiting stage in chemisorption, or different factors can have comparable contributions. At this point, it is crucial to understand to which extent kinetic parameters determined in batch for cryobeads are transferable to cryogel monoliths used under dynamic conditions. Several studies demonstrated better kinetics of sorption and catalysis on cryobeads in comparison with gel beads of the same composition in batch [25,26], but to the best of our knowledge there were no attempts to correlate sorption kinetics on cryobeads under static conditions with sorption properties of monoliths under dynamic conditions. However, development of the scalable method of cryogel fabrication is worth the effort only if the intrinsic properties of the sorption material are sufficiently good, and improvement of the mass transfer in column will significantly improve overall sorption performance under dynamic conditions.

Here, using the extended rate constants distribution (RCD) model [27] to determine Cu(II) ion sorption and desorption rates on cryobeads and gel beads of polyethyleneimine-based sorbent, we try to answer the question of whether we can use knowledge of sorption kinetic properties of cryobeads in batch to predict minimal efficient residence time of the adsorbate in monolith column of the same material. In addition, if yes, how do conditions of the kinetic experiments in batch affect the reliability of such predictions?

## 2. Results and Discussion

Tan and Hameed recently reviewed kinetic models used for adsorption from solutions, stating that in ideal case the model should reveal the rate-limiting mechanism and be useful to extrapolate kinetic parameters to the operating conditions of interest [28]. Many simplified phenomenological descriptive models assume diffusion- [29] or reaction-controlled [30] sorption kinetics and widely used to fit experimental kinetic curves with a limited number of adjustable parameters often lacking physical meaning [28,31]. Kinetic curve can be also considered to be a sum of fragments with different limiting factors, so that a separate kinetic model is applied for each fragment [28,32,33]. However, in most cases at each sorption stage sorption rate is affected by several factors, which are difficult to separate [28,33,34]. Moreover, sorbent surface heterogeneity can complicate such artificial fragmentation of the kinetic curve.

The approach used here is based on the Langmuir kinetics model [35,36,37,38,39], which links well-developed theoretical description of the adsorption equilibrium and pseudo-first- (PFO) or pseudo-second-order (PSO) rate kinetics equations. Since the chemical equilibrium is a balance between forward (adsorption) and reverse (desorption) rates of reaction, the equilibrium and kinetic constants can be determined through complete kinetic measurements [35]. Azizian [40,41,42] has made significant contributions to the solution of the Langmuir rate equation and its relationship with the PFO and PSO models, including development of model for characterization of sorbent heterogeneity [42], which, however, oversimplifies the sorption process and assumes the presence of two types of centers with significant difference in sorption rate constants.

The critical analysis of several models based on Langmuir kinetics is given by Marczewski et al. in [43,44], who have stated that these models were focused on description of the general kinetic behavior and determination of only one rate constant, which included both sorption and desorption sages. This is a significant disadvantage for the prediction of sorption dynamics using kinetic parameters obtained in batch experiments, since fast sorption centers with low affinity can merge with the slow sorption centers with high affinity, if the complete kinetic curve is analyzed. The RCD model [45,46,47,48] allows identification of the “fast” and “slow” sorption sites as well as sites with different affinity to adsorbate with understanding that “fast” sites are not necessarily the most affine ones, and a description of the sorption kinetics in the full range of surface coverages is crucial to predict sorbent performance in real-life applications.

We have recently developed and verified the extended RCD model, which enables one to determine affinity, quantity, and distribution of the sorption sites in the space of constants of sorption and desorption rates for heterogeneous sorbents via calculation of the RCD functions using experimental data obtained by the batch method [27]. Although this model yields apparent constants of the sorption/desorption rates, it can be used to predict distribution of the adsorbate on sorption centers from different starting conditions (solid:liquid ratio, adsorbate concentration) at any time of the sorption process. The detailed description of the model is given in [27]; here we will describe briefly the main theoretical approach and modifications of the model to account for diffusion limitations in sorption of Cu(II) ions on supermacroporous cryobeads and non-porous gel beads of polyethyleneimine cross-linked with diglycidyl ether of 1,4-butandiol. We refer further to these materials as PEI cryobeads and PEI-gels, respectively.

### 2.1. Theory and Data Analysis

#### 2.1.1. Rate Constants Distribution (RCD) Model

Description of the sorption kinetics using the concept of rate constants (RC) and Langmuir kinetic model was performed assuming a continuous distribution of the sorption sites in a heterogeneous sorbent (RCD model). The approach is based on the following assumptions: (1) flow of the adsorbate from the bulk to the sorbent is proportional to the adsorbate concentration in solution and to the surface area with vacant sorption sites; (2) flow of the adsorbate from the surface to the bulk is proportional to the surface area with occupied sorption sites; (3) specific surface area occupied with sorption sites of the certain type is proportional to the content of such sites in the sorbent.

To describe kinetics of sorption, we have introduced the density function *q*(*k_s_*, *k_d_*, *τ*), which shows the distribution of the adsorbate on sorption sites with the rate constants (RC) of sorption (*k_s_*) and desorption (*k_d_*) at any time point (*τ*), and the density function *q^max^* (*k_s_*, *k_d_*), which shows the maximal content of the adsorbate (sorption capacity) for the certain type of sorption sites (*k_s_*, *k_d_*) at full saturation, and *Q*^max^ is the total sorption capacity. Using these functions, the well-known Langmuir kinetic model equation for a homogeneous sorbent (1) will transform to Equation (2):

(1) $$\frac{dQ\left(\tau \right)}{d\tau }=k_{s}C\left(\tau \right)\left(Q^{\max}-Q\left(\tau \right)\right)-k_{d}Q\left(\tau \right)$$

(2) $$\frac{dq\left(k_{s},k_{d},\tau \right)}{d\tau }=k_{s}C\left(\tau \right)\left(q^{\max}\left(k_{s},k_{d}\right)-q\left(k_{s},k_{d},\tau \right)\right)-k_{d}q\left(k_{s},k_{d},\tau \right)$$

where *Q(τ)* is the total content of the adsorbate in the sorbent at time *τ*.

For complete description of the system equation of material balance (3) must be added to Equations (1) and (2):

(3) $$Q^{0}+V_{sp}\cdot C^{0}=Q\left(\tau \right)+V_{sp}\cdot C\left(\tau \right)$$

where *Q*^0^ is adsorbate content in the sorbent, *C*^0^ is adsorbate concentration in the solution in the initial time point; *V_sp_* is the specific solution volume. For the heterogeneous sorbents we can write the following integral equations:

(4) $$\begin{array}{l} \int_{0}^{+\infty }\int_{0}^{+\infty }q\left(k_{s},k_{d},\tau \right)dk_{s}dk_{d}=Q\left(\tau \right)\\ \int_{0}^{+\infty }\int_{0}^{+\infty }q^{0}\left(k_{s},k_{d},0\right)dk_{s}dk_{d}=Q^{0} \end{array}$$

Then the following Equation (5) can be written for the Langmuir-type sorption isotherm on a heterogeneous sorbent:

(5) $$Q^{e}=\int_{0}^{+\infty }\int_{0}^{+\infty }q^{\max}\left(k_{s},k_{d}\right)\frac{C^{e}k_{s}}{C^{e}k_{s}+k_{d}}dk_{s}dk_{d}$$

where *C^e^* and *Q^e^* are the equilibrium concentration of the adsorbate in the solution and the content of the adsorbate in the sorbent, respectively.

Transformation of the systems of integro-differential Equations (3) and (4) to the form suitable for numerical calculations and processing the experimental data are described in [27]. Obviously, there is no physical sense to assume that constants of the sorption/desorption rates can vary from −∞ to +∞; thus, we have limited the values of *K_s_* = log(*k_s_*) and *K_d_* = log(*k_d_*) to the reasonable range from −8 to +1. We have also introduced a grid for the RC space (*K_s_*, *K_d_*) with uniform splitting by each coordinate to n nodes. In preliminary calculations, *n* was varied from 9 to 100, and *n* = 20 was found to be the optimal value, which was further used in all calculations. In [27] we have used a regularization parameter to exclude sites with very high *q*_max_ value, if they did not significantly affect the kinetic curve. Here we have introduced the second regularization parameter to limit the minimal value of the desorption rate constant for highly affine sites (centers with *K_d_* [min^−1^] <−5,), so that *K_d_* value did not decrease until the minimum (*K_d_* = −8) during calculations, if it did not notably affect the kinetic curve. This limitation is required to avoid overestimation of the affinity constant *K_s_*/*K_d_*, since the accuracy of the adsorbate concentration determination is also limited and sorption on the centers with *K_d_* < −5 can be considered to be irreversible.

When the RCD function describing several kinetic curves for different solid:liquid ratios or starting concentrations of the adsorbate is found, the following density functions can be calculated to characterize the sorbent properties:

- 3D distribution of sorption sites in the space of constants of sorption/desorption rates—*ρ*(*K_s_*, *K_d_*);
- 2D distribution of sorption sites over constants of sorption rate (*K_s_* − *ρ*(*K_s_*));
- 2D distribution of sorption sites over constants of desorption rate (*K_d_* − *ρ*(*K_d_*));
- 2D affinity distribution of sorption sites over affinity constants (*K_AF_* = *K_s_*/*K_d_* − *ρ*(*K_AF_*)).

#### 2.1.2. Rate Constants Distribution (RCD)-Diffusion Model

A comprehensive overview of sorption diffusion models and range of their applicability is given by Tan [28]. Marczewski et al. discussed different approaches to include diffusion in Langmuir kinetics models with conclusions that results and range of applicability significantly depend on the kinetics equation used [44]. The sorption and desorption constants rates determined in the frame of the RCD model indirectly include diffusion contribution, since the model gives apparent kinetics parameters. Using kinetic curves obtained in a wide range of experimental conditions to obtain one RCD function increases reliability of the parameters, but if the bead size, stirring rate, flow direction and rate were changed, one can expect a change of diffusion contribution to the sorption rate. It is important to emphasize that calculation of the diffusion coefficients is not the primary tasks of adsorption–diffusion models; the most crucial is the ability to predict sorption kinetics under real operating conditions. Thus, here we focus on accounting diffusion in static experiments to the extent, which is necessary to predict sorption dynamics in monolith cryogels with minimal diffusion limitations. The RCD-diffusion model was extended using Fick’s second law for intraparticle diffusion, which is used in the most popular Crank diffusion model.

To construct the difference scheme for numerical solutions of the equation of the diffusion transfer from the finite volume of the solution inside the porous bead, the following starting conditions are required: solution volume (*V*), the initial concentration of the adsorbate in solution (*C_e_*^(0)^), the sorbent weight (*m_s_*), and the polymer phase does not contain adsorbate. The average bead radius was denoted as *r_b_* and the bead volume was divided to *n_d_* concentric diffusion layers with an equal increment ∆*r* = *r_b_*/*n_d_* and a coordinate origin set to the bead center. Let us denote the swelling degree of the sorbents by *ε* (*ε* = *m_b_*/*m_s_* × 100% =((*m_w_* + m_s_)/*m_s_*)·100%, where *m_b_*—the weight of the swollen beads, *m_w_*—the weight of the water in beads; the distance from the bead center to the inner surface of the i-diffusion layer as *r_i_* = *i*·∆*r* (the surface, which is closer to the bead center, was assumed as the inner surface); the specific volume of the solution as *V_sp_* = *V/m_s_*, the water density as *ρ_w_*, the diffusion coefficient as *D*, the time increment of the diffusion transfer as ∆*t_d_*, and the specific volume of the solution in diffusion layers as *V_Lsp_* (*V_Lsp_* = 1/ *ρ_w_* × (ε/100 − 1).

Using Fick’s law and the material balance condition for the diffusion from finite solution volume for this system, we can obtain the following difference scheme between the adsorbate concentrations at *τ*_*k*_ and *τ*_*k*+1_:

(6) $$\Delta \tau_{d}\leq \frac{\tau^{*}}{6n_{d}^{2}}$$

(7) $$\begin{array}{l} c_{0}^{\left(k+1\right)}=\left(1-3n_{d}^{2}\frac{\Delta \tau_{d}}{\tau^{*}}\right)c_{0}^{\left(k\right)}+3n_{d}^{2}\frac{\Delta \tau_{d}}{\tau^{*}}c_{1}^{\left(k\right)}\\ c_{i}^{\left(k+1\right)}=\left(1-3n_{d}^{2}\frac{\Delta \tau_{d}}{\tau^{*}}\frac{2i^{2}+2i+1}{3i^{2}+3i+1}\right)c_{i}^{\left(k\right)}+\\ +3n_{d}^{2}\frac{\Delta \tau_{d}}{\tau^{*}}\frac{i^{2}+2i+1}{3i^{2}+3i+1}c_{i+1}^{\left(k\right)}+3n_{d}^{2}\frac{\Delta \tau_{d}}{\tau^{*}}\frac{i^{2}}{3i^{2}+3i+1}c_{i-1}^{\left(k\right)};1\leq i\leq n_{d}-2\\ c_{n_{d}-1}^{\left(k+1\right)}=\left(1-3n_{d}^{2}\frac{\Delta \tau_{d}}{\tau^{*}}\frac{2n_{d}{}^{2}-2n_{d}+1}{3n_{d}{}^{2}-3n_{d}+1}\right)c_{n_{d}-1}^{\left(k\right)}+\\ +3n_{d}^{2}\frac{\Delta \tau_{d}}{\tau^{*}}\frac{n_{d}{}^{2}}{3n_{d}{}^{2}-3n_{d}+1}c_{e}^{\left(k\right)}+3n_{d}^{2}\frac{\Delta \tau_{d}}{\tau^{*}}\frac{n_{d}{}^{2}-2n_{d}+1}{3n_{d}{}^{2}-3n_{d}+1}c_{n_{d}-2}^{\left(k\right)}\\ c_{e}^{\left(k+1\right)}=\left(1-3n_{d}\frac{\Delta \tau_{d}}{\tau^{*}}\frac{V_{Lsp}}{V_{sp}}\right)c_{e}^{\left(k\right)}+3n_{d}\frac{\Delta \tau_{d}}{\tau^{*}}\frac{V_{Lsp}}{V_{sp}}c_{n_{d}-1}^{\left(k\right)} \end{array}$$

where τ* is the “diffusion characteristic time” (τ* = *r_b_*^2^/*D*); *C_i_*^(*k*)^ is the adsorbate concentration in the i-diffusion layer at the time *τ*_*k*_. Inequality (6) limits the maximal time increment of the diffusion transfer to assure stability of the difference scheme.

As one can see from Equation (7), it is impossible to determine the bead radius and the diffusion coefficient simultaneously from sorption kinetic curves, but the values of “characteristic time” of diffusion can be determined for the given kinetic curve. On the other hand, for the systems, which vary only in the average bead radius, one can determine the relative average radius as a square root of the “characteristic times” ratio.

In the RCD-diffusion model, each step of simulating the sorption kinetics curve, taking into account the intraparticle diffusion, is implemented in two steps: step #1—simulation of intraparticle diffusion; step #2—simulation of sorption kinetics using the RCD functions model [27] in each diffusion layer. Let us denote the time increment for simulation of sorption kinetics as ∆*τ_s_*; then, if at the simulation time *τ* the value of ∆*τ_d_* calculated using the Equation (6) is larger than ∆*τ_s_*, step #1 is executed for the time interval ∆*τ_s_* followed by execution of the step #2. In opposite case, step #1 is executed several times for the time intervals ∆*τ_d_*(*τ^k^*), until *τ*^*k*^≤ *τ* + ∆*τ_s_*, where:

(8) $$\begin{array}{l} \tau^{\left(k+1\right)}=\tau^{\left(k\right)}+\Delta \tau_{d}\left(\tau^{\left(k\right)}\right)\\ \tau^{\left(0\right)}=\tau \end{array}$$

Then, step #2 shall be executed for the time interval ∆*τ_s_*. When the RCD diffusion model is applied to the kinetic curves obtained at different conditions (solid:liquid ratio, initial adsorbate concentration) for the sorbent with one bead size “characteristic time” of diffusion must be considered to be another variable allowing better description of the experimental data set with one RCD function. To obtain the values of the characteristic time of diffusion, which have the physical sense of diffusion coefficient, the RCD-diffusion model must be applied to the sets of kinetic curves obtained for the same sorbent with different bead sizes. It must be taken into account that the constant bead size through the whole kinetic experiment is the crucial factor. Taking into account poor mechanical stability of cryobeads, the bead size variation in the broad range is not realistic for most systems, including PEI cryobeads reported here.

### 2.2. Application of Phenomenological PFO and PSO Models to Cu(II) Sorption Kinetics on PEI Cryobeads and Gels

As mentioned in the introduction, most works on comparison of sorption kinetics on cryobeads in gel beads show improvement due to the porous structure but, in most cases, kinetics is rather slow, allowing the reaching of >90% of the adsorbate recovery in the timespan of 30–60 min [15,25], which is much longer than the adsorbate residence time expected for efficient sorbents in column applications. In our recent work [27], we have suggested that stagnation zones formed in the swollen cryobeads can significantly slow down sorption kinetics even at high fluid shear, if the flow does not go through the cryobeads in the same way as it goes through the 3D porous structure of the monolith under dynamic conditions.

**Figure 1:**
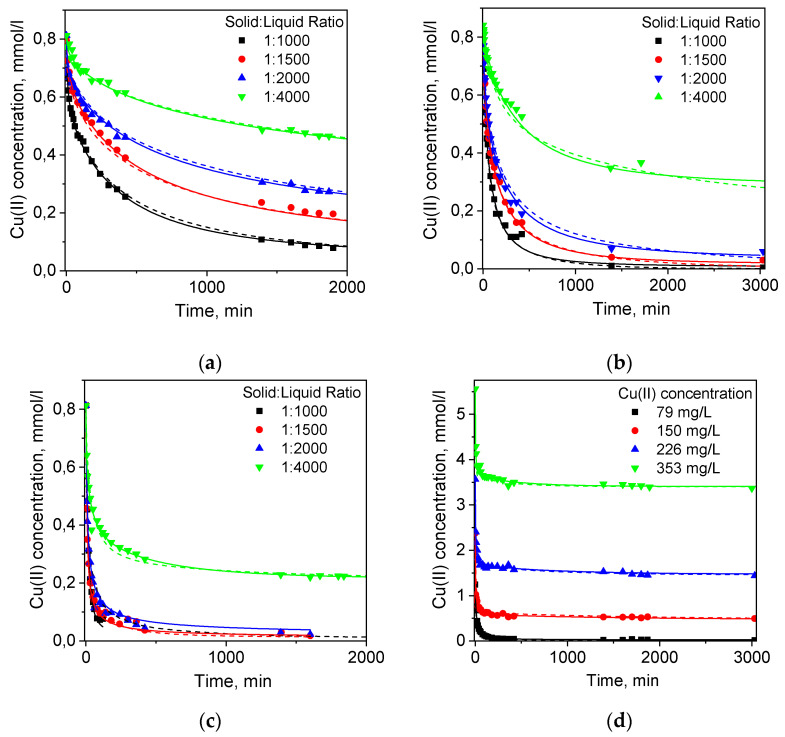
Kinetic curves of Cu(II) ion sorption from 1 M NH_4_OAc solution on the (**a**) PEI gel beads; (**b**) PEI cryobeads; (**c**) PEI gel fines); (**d**) PEI cryobeads fines. Dots—experimental data; dashed lines—RCD model; solid lines—RCD-diffusion model.

Here we have compared the kinetics of Cu(II) ion sorption on porous cryobeads and non-porous gel beads of cross-linked PEI and fines of both sorbents (Figure 1) using PFO and PSO kinetic models, and extended RCD and RCD-diffusion models. The fines were used to reduce intraparticle diffusion limitations in sorption kinetics and compare effects of grinding and high porosity on the calculated kinetic parameters. We have found that the PSO model provided better fits to all kinetic curves (results of linearization kinetic curves and parameters of PFO and PSO models are shown in Supplementary Materials, Figure S1, Tables S1 and S2). Since phenomenological models describe sorption kinetics without separation of sorption stages—external film diffusion, chemical reaction, intraparticle diffusion, the determined in PSO and PFO models RC are the average values.

**Figure 2:**
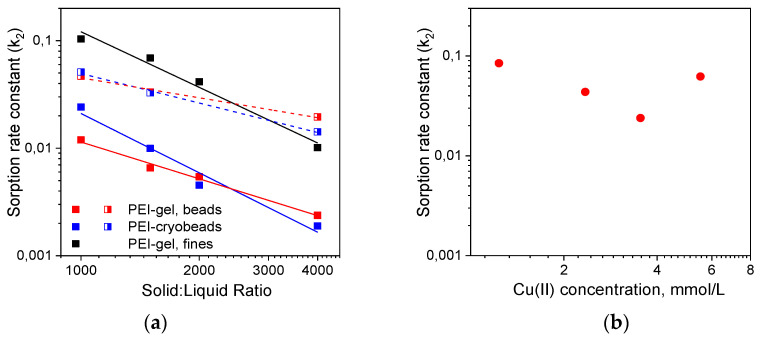
Constants of Cu(II) ions sorption rate (g·mmol^−1^ min^−1^) on PEI-gels and PEI cryogel: (**a**) Cu(II) concentration 50 mg/L, linearization using PSO model was executed for the timespan 3000 min (closed symbols, solid lines) and in the timespan corresponding to Cu(II) adsorbed amount of 0.5 mmol/g (half-open symbols, dash lines); (**b**) Cu(II) sorption on PEI cryobeads at solid:liquid ratio 1:1000, linearization using PSO model was executed for the timespan 3000 min.

Figure 2a and Table S2 (Supplementary Materials) show that constants of Cu(II) sorption rates on PEI cryogel and PEI gel beads and fines depend on the solid:liquid ratio, initial adsorbate concentration, and the time interval used for linearization. The difference between sorption kinetics on PEI gel and PEI cryogel beads was not remarkable. If the PSO model was applied to the full kinetic curve, sorption RC were only 2–3-fold higher for PEI cryobeads.

When linearization was performed for the time intervals corresponding to Cu(II) adsorbed amounts of 0.5 mmol/g (~20% of the sorption capacity), this difference became negligible. At the same time, sorption RC on fines of PEI gel were 5–10 times higher than on beads of PEI gel and cryogel (Figure 2a, Table S2) showing that the porous structure of cryobeads does not significantly improve sorption kinetics under static conditions and diffusion limitations are still very important. Moreover, Figure 2b shows that there was no linear correlation between the adsorbate concentration and the sorption rate constant on PEI cryobeads at fixed solid:liquid ratio, which can be explained by interplay of different factors affecting average value of the RC calculated using PSO model. Thus, the application of the phenomenological PFO and PSO kinetic models to these experimental data can give sets of apparent sorption RC, without physicochemical significance and revealing adsorption mechanisms, which allowed comparison between materials but not prediction of the sorption kinetics at arbitrary chosen experimental conditions.

### 2.3. Application of the Extended RCD and RCD-Diffusion Models to Investigate Kinetics of Metal Ion Sorption on PEI Cryobeads and Gels

The RCD model is based on the assumption of the reversible adsorption process on several types of sorption centers that allows accounting for sorbent heterogeneity and calculation ofadsorbate distribution between different types of the sorption centers at different stages of sorption. Using the RCD model, one must process simultaneously several kinetic curves obtained at different sorption ratios and/or adsorbate concentrations to yield one RCD function, which in an ideal case describes the “intrinsic” properties of the sorbent and can be used to model sorption kinetics under arbitrary conditions.

**Figure 3:**
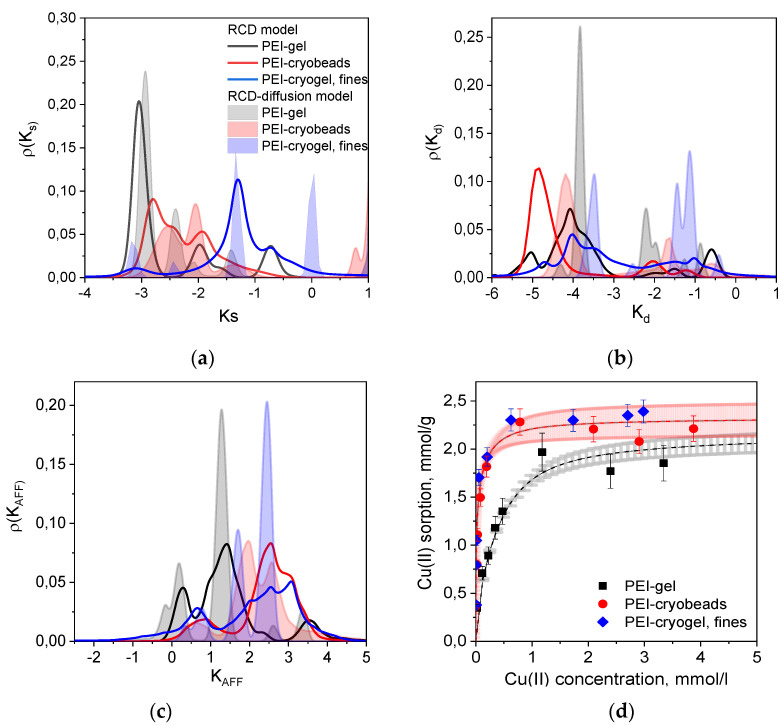
2D distribution functions for Cu(II) ions sorption centers of PEI-based materials over the constants of (**a**) sorption rates (*K_s_*), (**b**) desorption rates (*K_d_*), (**c**) affinity(*K_AFF_*). The legend in Figure 3a is valid for Figure 3b,c. (**d**) Isotherms of Cu(II) ions sorption on PEI-based materials, dots—experimental data; dashed lines with confidence intervals (color-filled areas)—isotherms calculated using RCD model: grey—PEI gel beads; red—PEI cryobeads.

Figure 3 shows several types of distribution functions calculated from the RCD functions found for PEI gel, PEI cryobeads, and fines of PEI cryogel from the kinetic curves depicted in Figure 1. The RCD model confirms general trend, which was found by applying the PSO model: the Cu(II) sorption rate constants on beads of PEI gel and PEI cryogel differ much less in comparison with fast sorption on fines of PEI cryogel. Comparison of the distribution function for the sorption (Figure 3a) and desorption (Figure 3b) rates constants shows that regardless of the porosity and the bead size several types of sorption centers can be identified. Slow sorption centers with *K_s_* (log*k_s_*) < −2.5, which dominate for PEI gel beads, were also found for fines of PEI cryogel. Distribution functions for constants of desorption rates (*K_d_*) clearly show the presence of two types of sorption centers, which can be denoted as low affinity (high *K_d_*) and high affinity (low *K_d_*). To simplify the comparison between kinetic properties of the materials, the weighted average sorption and desorption RC were calculated and summarized in Table 1. Since “fast” sorption sites with high *K_s_* can be of low or high affinity, the affinity constant (*K_AFF_* = *K_s_*/*K_d_*) distribution function (Figure 3c) provides better visualization of the sorption centers characteristics. Diffusion limitations affect both sorption and desorption rates; therefore, the differences between the affinity constants for PEI gel, cryogel, and fines becomes significantly lower than can be assumed from *K_s_*-distribution functions for these materials. Since the RCD function provides complete information on sorption capacities and affinities of sorption centers, it can be used to calculate full theoretical sorption isotherms using Equation (3). Figure 3d shows very good correlation between experimental and calculated isotherm of Cu(II) ion sorption on PEI gel, cryogel, and fines. Since the isotherm corresponds to the equilibrium state of sorption, no difference was observed for PEI cryobeads and PEI cryogel fines. PEI gel has lower sorption capacity due to higher cross-linking degree at room temperature.

**Figure 4:**
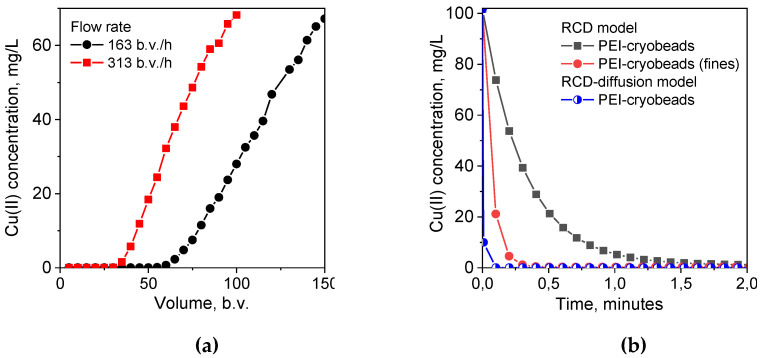
(**a**). The breakthrough curves of Cu(II) ions sorption on monolith PEI cryogel from 1 M NH_4_OAc solution; pH 5.3; Cu—100 mg/L; monolith volume—1 mL. (**b**) Simulated kinetic curves for Cu(II) ions sorption on monolith PEI cryogel using RCD functions calculated for Cu(II) sorption on PEI cryogel beads and fines, parameters for simulation: dry sorbent weight—0.085 g; solution volume—1 mL; Cu(II) concentration—100 mg/L.

As we have mentioned above, the important advantage of the RCD model is the possibility to predict sorption kinetics at any arbitrary conditions, including sorption at low solid:liquid ratio corresponding to the sorption dynamics on monolith cryogel in column, using RCD functions calculated from experimental kinetic curves. In this case, simulated kinetic curves can be used to determine the minimal residence time of solution in sorption column required to reach certain degree of the adsorbate uptake. The experimental breakthrough curves for Cu(II) ion sorption on monolith PEI cryogel (Figure 4a) show that ~100% uptake of Cu(II) ions was achieved at very high flow rates—163 bed volume (b.v.)/h and 313 b.v./h corresponding to the residence time of the adsorbate in the column of 0.35 min and 0.19 min, respectively. However, simulated kinetic curves for Cu(II) ion sorption on PEI cryogel for the same sorption conditions as in column (solid:liquid ratio, sorbent weight, and Cu(II) concentration) showed that expected Cu(II) uptake would be only 65% for the flow rate of 163 b.v./h and 45% for 313 b.v./h, if PEI monolith had kinetic properties of PEI cryobeads (Figure 4b). This confirms serious diffusion limitation for the sorption on cryobeads in batch. The kinetic curve simulated using RCD function for sorption on fines of PEI cryogel gives significantly better prediction of the minimal Cu(II) residence time in column for ~100% uptake but still overestimates it in comparison with the experimental data.

(9) $$\begin{array}{l} K_{s}=\frac{1}{Q_{\max}}\sum_{i}^{}q_{\max,i}\cdot \left(K_{s,}{}_{i}\right)\\ K_{d}=\frac{1}{Q_{\max}}\sum_{i}^{}q_{\max}{{}_{,}}_{i}\cdot \left(K_{d,}{}_{i}\right) \end{array}$$

where *K_s_*, and *K_d_*—the logarithms of weighted average sorption and desorption RC, respectively, *Q*_max_—the maximum sorption capacity; *K_s,i_/K_d,i_* and *q_i_*—the logarithms of the sorption and desorption RC and sorption capacity for the i-type of the sorption center.

As mentioned above, the important advantage of the RCD model is the possibility to predict sorption kinetics at any arbitrary conditions, including sorption at low solid:liquid ratio corresponding to the sorption dynamics on monolith cryogel in column, using RCD functions calculated from experimental kinetic curves. In this case, simulated kinetic curves can be used to determine the minimal residence time of solution in a sorption column required to reach certain degree of the adsorbate uptake. The experimental breakthrough curves for Cu(II) ion sorption on monolith PEI cryogel (Figure 4a) show that 100% uptake of Cu(II) ions was achieved at very high flow rates—163 bed volume (b.v.)/h and 313 b.v./h corresponding to the residence time of the adsorbate in the column of 0.35 min and 0.19 min, respectively. However, simulated kinetic curves for Cu(II) ion sorption on PEI cryogel for the same sorption conditions as in column (solid:liquid ratio, sorbent weight, and Cu(II) concentration) showed that expected Cu(II) uptake would be only 65% for the flow rate of 163 b.v./h and 45% for 313 b.v./h, if PEI monolith had kinetic properties of PEI cryobeads (Figure 4b). This confirms serious diffusion limitation for the sorption on cryobeads in batch. The kinetic curve simulated using RCD function for sorption on fines of PEI cryogel gives significantly better prediction of the minimal Cu(II) residence time in column for 100% uptake but still overestimates it in comparison with the experimental data. Taking into account the weighted average sorption RC for PEI cryobeads and fines (Table 1), we can conclude that the sorption rate on monolith is more than ten-fold higher than on PEI cryobeads, and sorption on fines is still limited by intraparticle diffusion.

To obtain the RCD function, which describes “intrinsic” kinetic properties of the sorbent, one must eliminate diffusion contributions. Using the approach described in Section 2.1.2, we have applied the RCD-diffusion model to the kinetic curves depicted in Figure 1 and calculated corresponding distribution functions (Figure 3a–c, Table 1). First, introduction of the “characteristic time” of diffusion as another variable parameter resulted in the decrease of residual dispersion (Table 1) and allowed better description of kinetic curves in each data sets (Figure 1). Although the RCD diffusion model showed the same trends in kinetic properties of beads and fines of PEI gel and cryogel, the maxima of the *K_s_*- and *K_d_*-distribution functions were shifted to the higher values for ~1 log unit. Application of the RCD-diffusion model to process jointly two datasets for Cu(II) sorption on PEI cryogel beads and fines confirmed that one RCD function can be found to describe sorption kinetics on materials, which have the same chemical structure but different particle size (Table 1). The “characteristic time” of diffusion for fines of PEI cryogel was more than ten-fold lower than that for PEI cryobeads, emphasizing the role of diffusion limitation in sorption on cryobeads. Using the RCD diffusion function for PEI cryogel for simulation of the kinetic curve, we have calculated the minimal residence time of Cu(II) in a column for efficient uptake as 0.09 min (Figure 4b), which is in good agreement with experimental breakthrough curves.

It is not surprising, taking into account high swelling degree of cryogels (up to several thousand %) and presence of not only free (“squeezable”) water in macropores but also water bound to the polymer. This structure of the swollen cryobeads assumes the existence of the stagnation zones inside cryobeads, where mass transfer is hindered even at high rates of external stirring. Under dynamic conditions, high efficiency of mass transfer is guaranteed by constant flow but if the limiting stage of the sorption process is the chemical reaction, one cannot benefit from morphology of the cryogel. That is why we believe that it is crucial to distinguish between physical (diffusion) and chemical (reaction rate) limitations at the first stage of the development of cryogels for versatile applications and perform kinetic investigations in batch under appropriate conditions.

## 3. Conclusions

Here we have suggested the extended RCD model accounting for intraparticle diffusion and applied it to investigation of Cu(II) ions sorption kinetics on swollen porous (cryobeads) and non-porous (gel beads) and fines of cross-linked polyethyleneimine. We have found that:

1. Sorption rate on highly swollen cryobeads in batch is only 2–3 times higher than on gel beads, while sorption rates on fines are 5–10 times higher than on beads. Thus, macroporosity does not eliminate diffusion limitation in batch experiments, only insignificantly reduces them.
2. RCD model shows the existence of “slow” and “fast” sorption sites regardless of the particle size. Since diffusion limitations affect both rates of sorption and desorption, affinity distribution functions for sorption of Cu(II) ions on cryobeads and fines are in good correlation. Constants of sorption/desorption rates calculated using RCD-diffusion model are ~1 log unit higher than constants determined with the RCD model.
3. Kinetic parameters calculated in the RCD-diffusion model for fines and cryobeads of PEI can be used to predict residence time required for efficient uptake of the adsorbate in column under dynamic conditions. Parameters calculated from the RCD model have yielded overestimated minimal residence time.

Thus, the reliable research protocols to study the kinetics of sorption on cryogels must be developed. To reveal benefits of the cryogel structure for application as monoliths, sorption performance must be investigated under dynamic conditions. If kinetic properties are investigated for cryobeads in batch, one has to account for the diffusion using adequate kinetic models or particle size of cryogels has to be reduced in the same way as it is done in investigation of non-porous sorbents.

## 4. Materials and Methods

### 4.1. Materials

Branched polyethylenimine (PEI) with the average molecular weight of 25 kDa was purchased from “AlfaAesar”. 1,4-butanediol diglycidyl ether was purchased from Sigma–Aldrich. Other reagents were of analytical grade.

### 4.2. PEI Cryogels and Gels Fabrication

**Figure 5:**
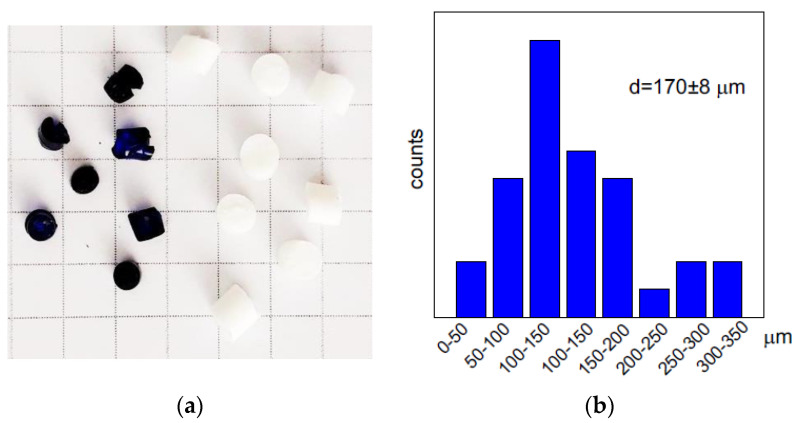
Photo of PEI gel-beads (blue beads after Cu(II) sorption) and PEI cryobeads (white, before sorption), cell size in photo 0.5 × 0.5 cm (**a**). Particle size distribution for fines of PEI cryobeads (**b**).

PEI was cross-linked in 5% solution (pH 10) with 1,4-butanediol diglycidyl ether at molar ratio to PEI (monomer unit) 1:4 as described in detail in [27]. To fabricate monolith PEI cryogel, a PEI solution with cross-linker agent was placed into insulin syringes with inner diameter of 4.8 mm and kept frozen at −20 °C for 7 days. After thawing, the monolith cryogels were washed with distilled water using a peristaltic pump (Ismatec, Wertheim, Germany) to remove unreacted chemicals and sealed in the swollen state until used. To obtain cylindrically shaped beads, after addition of the cross-linker, PEI solutions were placed into plastic tubes of the inner diameter of 3.6 mm and kept frozen at −20 °C for 7 days for PEI cryobeads or at room temperature for PEI gel. Cross-linked PEI cryogels and gels were removed from the plastic tubes, washed with deionized water, cut to the cylinders of a length of ~3 mm, and dried at room temperature. Swelling of the cross-linked PEI cryobeads and gel beads was determined from the difference in weights of the swollen for 24 h in distilled water and dry material—1700% and 300%, respectively. After re-swelling during sorption cryobeads completely recovered the size of beads, while dried gel beads slightly shrank. Figure 5a shows the photo of PEI cryobeads and gel beads with average size 0.35 ± 0.03 cm and 0.26 ± 0.02 cm, respectively. The cryobeads fines were obtained by ultrasound treatment of the swollen PEI cryobeads and characterized using optical microscopy and ImageJ software [49] to calculate the particle size distribution (Figure 5b). The structure of the swollen (never dried) and stained with fluorescein PEI cryogels was investigated using a Carl Zeiss LSM 780 confocal laser scanning microscope (Oberkochen, Germany), the average pore size was 128 ± 30 μm (Figure S2, Supplementary Materials).

### 4.3. Investigations of the Sorption Properties of PEI Cryobeads, PEI Gel, and Monolith PEI Cryogel

The kinetics of Cu(II) sorption on PEI cryobeads and PEI gel at different sorbent:solution ratios (1:1000–1:4000) was studied from ammonia acetate solution (1 mol/L) containing Cu(NO_3_)_2_ at 23 °C, pH = 5.01 ± 0.01, initial Cu(II) concentration was 50 mg/L. The kinetics of Cu(II) sorption on fines of PEI cryobeads was studied at sorbent:solution ratio 1:1000 at various initial Cu(II) concentrations—79 mg/L, 150 mg/L, 226 mg/L, and 353 mg/L. The cryobeads, gels, and fines were placed into the large permeable for solution closed bags, which were inert toward Cu(II) ions sorption, and constantly agitated using a Biosan PSU−20i orbital shaker (Riga, Latvia) at 290 rpm, the sampling of the solution was performed regularly during 3000 min. The copper concentration in the solutions was determined by the atomic absorption spectrometry (AAS) using a Solaar M6 (Thermo Scientific, Waltham, MA, USA) device.

The sorption isotherms were investigated from ammonia acetate solution (1 mol/L) at the sorbent:solution ratio 1:1000, the contact time was 3000 min, the solutions with sorbents were agitated under the same conditions as in kinetics study. At least three replicates were made to assure the results reproducibility. The adsorbed amounts were calculated using the difference in initial and equilibrium concentrations of the metal ions in the solutions determined by AAS.

Dynamics of Cu(II) sorption on monolith PEI cryogel was investigated as follows: solution of ammonia acetate (1 mol/L) containing 100 mg/L of Cu(II) in the form of Cu(NO_3_)_2_ was fed through a syringe with 1 mL of the swollen cryogel (inner diameter—4.8 mm, bed length—6 cm) at flow rates of 163 and 313 b.v./h. The samples were collected every 5 mL, copper concentrations were determined with AAS.

## Figures and Tables

**Table 1:** The parameters of continuous RCD and RCD-diffusion (RCD-D) models for kinetic curves of Cu(II) ions sorption on PEI cryogels and gels.

Sorbent	Τ ^1^	Residual Dispersion (×10^4^)		*K_s_* ^3^		*K_d_* ^3^		Q_max_, mmol/g ^2^	
		RCD-D	RCD	RCD-D	RCD	RCD-D	RCD	RCD-D	RCD
PEI-cryobead		4.5	7.4	−1.66	−2.34	−3.51	−4.43	2.31	2.36
PEI cryogel, fines		1.0	14	−0.50	−1.36	−2.10	−3.13	2.13	2.19
PEI gel		2.1	5.2	−2.39	−2.47	−3.12	−3.42	2.41	2.37
PEI gel, fines		2.8	34	−1.13	−1.04	−3.03	−2.08	2.45	2.37
**Application to datasets with different particle sizes**									
PEI-cryobead PEI cryogel, fines	19.4 1.84	8.9		−0.50		−2.20		2.10	

^1^—“characteristic time” of diffusion (min), ^2^—the equilibrium value for the infinite period of sorption time, ^3^—the weighted average sorption and desorption rate constants calculated according to Equation (9).

## Supplementary Materials

The following are available online at https://www.mdpi.com/2310-2861/6/2/15/s1, Figure S1: Linear PFO model for Cu(II) ions adsorption kinetics of PEI-gels and PEI cryobeads, Figure S2. Confocal laser scanning microscopy (CLSM) images of PEI-cryogels stained with fluorescein, Table S1. The parameters of PFO model for kinetic curves of Cu(II) ions sorption on PEI cryogels and gels—k1 is rate constant of pseudo-first-order adsorption, Qe is Cu(II) adsorbed amounts at quasi-equilibrium, Table S2. The parameters of PSO model for kinetic curves of Cu(II) ions sorption on PEI cryogels and gels—k2 is rate constant of pseudo-second-order adsorption, Qe is Cu(II) adsorbed amounts at quasi-equilibrium.

## References

[1] Lozinsky V. (2018). Cryostructuring of Polymeric Systems. 50.† Cryogels and Cryotropic Gel-Formation: Terms and Definitions. Gels.

[2] Baimenov A., Berillo D.A., Poulopoulos S.G., Inglezakis V.J. (2020). A review of cryogels synthesis, characterization and applications on the removal of heavy metals from aqueous solutions. Adv. Colloid Interface Sci..

[3] Hixon K.R., Lu T., Sell S.A. (2017). A comprehensive review of cryogels and their roles in tissue engineering applications. Acta Biomater..

[4] Savina I.N., Ingavle G.C., Cundy A.B., Mikhalovsky S.V. (2016). A simple method for the production of large volume 3D macroporous hydrogels for advanced biotechnological, medical and environmental applications. Sci. Rep..

[5] Önnby L. (2016). Application of cryogels in water and wastewater treatment. Supermacroporous Cryogels Biomed. Biotechnol. Appl..

[6] Lozinsky V.I. (2008). Polymeric cryogels as a new family of macroporous and supermacroporous materials for biotechnological purposes. Russ. Chem. Bull..

[7] Kumar A., Srivastava A. (2010). Cell separation using cryogel-based affinity chromatography. Nat. Protoc..

[8] Pan M., Shen S., Chen L., Dai B., Xu L., Yun J., Yao K., Lin D.Q., Yao S.J. (2015). Separation of lactoperoxidase from bovine whey milk by cation exchange composite cryogel embedded macroporous cellulose beads. Sep. Purif. Technol..

[9] Plieva F.M., Seta E.D., Galaev I.Y., Mattiasson B. (2009). Macroporous elastic polyacrylamide monolith columns: Processing under compression and scale-up. Sep. Purif. Technol..

[10] Dragan E.S., Humelnicu D., Dinu M.V. (2019). Development of chitosan-poly(ethyleneimine)-based double network cryogels and their application as superadsorbents for phosphate. Carbohydr. Polym..

[11] Dragan E.S., Loghin D.F.A. (2018). Fabrication and characterization of composite cryobeads based on chitosan and starches-g-PAN as efficient and reusable biosorbents for removal of Cu2+, Ni2+, and Co2+ ions. Int. J. Biol. Macromol..

[12] Dragan E.S., Lazar M.M., Dinu M.V., Doroftei F. (2012). Macroporous composite IPN hydrogels based on poly(acrylamide) and chitosan with tuned swelling and sorption of cationic dyes. Chem. Eng. J..

[13] Dobritoiu R., Patachia S. (2013). A study of dyes sorption on biobased cryogels. Appl. Surf. Sci..

[14] Apopei D.F., Dinu M.V., Trochimczuk A.W., Dragan E.S. (2012). Sorption isotherms of heavy metal ions onto semi-interpenetrating polymer network cryogels based on polyacrylamide and anionically modified potato starch. Ind. Eng. Chem. Res..

[15] Jalilzadeh M., Uzun L., Serap S. (2016). Specific heavy metal ion recovery with ion-imprinted cryogels. J. Appl. Polym. Sci..

[16] Onnby L., Giorgi C., Plieva F.M., Mattiasson B. (2010). Removal of heavy metals from water effluents using supermacroporous metal chelating cryogels. Biotechnol. Prog..

[17] Hajizadeh S., Kirsebom H., Galaev I.Y., Mattiasson B. (2010). Evaluation of selective composite cryogel for bromate removal from drinking water. J. Sep. Sci..

[18] Sahiner N., Demirci S. (2016). Poly ionic liquid cryogel of polyethyleneimine: Synthesis, characterization, and testing in absorption studies. J. Appl. Polym. Sci..

[19] Shu Y., Huang R., Wei X., Liu L., Jia Z. (2017). Pb(II) Removal Using TiO_2_-Embedded Monolith Composite Cryogel as an Alternative Wastewater Treatment Method. Water. Air. Soil Pollut..

[20] Kil’deeva N.R., Veleshko I.E., Vladimirov L.V., Nikonorov V.V., Lozinskii V.I., Ivanov R.V., Perminov P.A., Mikhailov S.N. (2012). Modification of chitosan cryogels by pyridoxal phosphate to improve sorption capacity. Fibre Chem..

[21] Sahiner N. (2018). Super macroporous poly(N-isopropyl acrylamide) cryogel for separation purpose. Polym. Adv. Technol..

[22] Lozinsky V.I., Galaev I.Y., Plieva F.M., Savina I.N., Jungvid H., Mattiasson B. (2003). Polymeric cryogels as promising materials of biotechnological interest. Trends Biotechnol..

[23] Aşir S., Uzun L., Türkmen D., Say R., Denizli A. (2005). Ion-selective imprinted superporous monolith for cadmium removal from human plasma. Sep. Sci. Technol..

[24] Alkan H. (2015). Poly(hydroxyethyl methacrylate)-co-N-methacryloyl-(L)-histidine methyl ester Based Cryogel for the Removal of Fe3+ from Human Plasma effected with Beta Thalassemia. Hacettepe J. Biol. Chem..

[25] Privar Y., Malakhova I., Pestov A., Fedorets A., Azarova Y., Bratskaya S. (2018). Polyethyleneimine cryogels for metal ions sorption. Chem. Eng. J..

[26] Sahiner N., Demirci S. (2016). PEI-based hydrogels with different morphology and sizes: Bulkgel, microgel, and cryogel for catalytic energy and environmental catalytic applications. Eur. Polym. J..

[27] Golikov A., Malakhova I., Azarova Y., Eliseikina M., Privar Y., Bratskaya S. (2020). Extended Rate Constant Distribution Model for Sorption in Heterogeneous Systems. 1: Application to Kinetics of Metal Ion Sorption on Polyethyleneimine. Cryogels. Ind. Eng. Chem. Res..

[28] Tan K.L., Hameed B.H. (2017). Insight into the adsorption kinetics models for the removal of contaminants from aqueous solutions. J. Taiwan Inst. Chem. Eng..

[29] Weber W.J., Morris J.C., Sanit J. (1963). Kinetics of Adsorption on Carbon from Solution. J. Sanit. Eng. Div. Am. Soc. Civ. Eng..

[30] Lagergren S. (1898). Zur Theorie der Sogenannten Adsorption Geloster Stoffe. K. Sven. Vetenskakad. Handl..

[31] Alberti G., Amendola V., Pesavento M., Biesuz R. (2012). Beyond the synthesis of novel solid phases: Review of modelling of sorption phenomena. Coord. Chem. Rev..

[32] Malash G.F., El-Khaiary M.I. (2010). Piecewise linear regression: A statistical method for the analysis of experimental adsorption data by the intraparticle-diffusion models. Chem. Eng. J..

[33] Douven S., Paez C.A., Gommes C.J. (2015). The range of validity of sorption kinetic models. J. Colloid Interface Sci..

[34] Hu Q., Xie Y., Feng C., Zhang Z. (2019). Fractal-like kinetics of adsorption on heterogeneous surfaces in the fixed-bed column. Chem. Eng. J..

[35] Kuan W.H., Lo S.L., Chang C.M., Wang M.K. (2000). A geometric approach to determine adsorption and desorption kinetic constants. Chemosphere.

[36] Novak L.T., Adriano D.C. (1975). Phosphorus Movement in Soils: Soil-Orthophosphate Reaction Kinetics1. J. Environ. Qual..

[37] Liu Y., Shen L. (2008). From Langmuir kinetics to first- and second-order rate equations for adsorption. Langmuir.

[38] Zhang J. (2019). Physical insights into kinetic models of adsorption. Sep. Purif. Technol..

[39] Salvestrini S. (2018). Analysis of the Langmuir rate equation in its differential and integrated form for adsorption processes and a comparison with the pseudo first and pseudo second order models. React. Kinet. Mech. Catal..

[40] Azizian S. (2004). Kinetic models of sorption: A theoretical analysis. J. Colloid Interface Sci..

[41] Azizian S., Haerifar M., Bashiri H. (2009). Adsorption of methyl violet onto granular activated carbon: Equilibrium, kinetics and modeling. Chem. Eng. J..

[42] Azizian S. (2006). A novel and simple method for finding the heterogeneity of adsorbents on the basis of adsorption kinetic data. J. Colloid Interface Sci..

[43] Marczewski A.W. (2010). Analysis of kinetic langmuir model. Part I: Integrated kinetic langmuir equation (IKL): A new complete analytical solution of the langmuir rate equation. Langmuir.

[44] Marczewski A.W., Deryło-Marczewska A., Słota A. (2013). Adsorption and desorption kinetics of benzene derivatives on mesoporous carbons. Adsorption.

[45] Choi H., Al-Abed S.R. (2009). PCB congener sorption to carbonaceous sediment components: Macroscopic comparison and characterization of sorption kinetics and mechanism. J. Hazard. Mater..

[46] Monazam E.R., Shadle L.J., Miller D.C., Pennline H.W., Fauth D.J., Hoffman J.S., Gray M.L. (2013). Equilibrium and kinetics analysis of carbon dioxide capture using immobilized amine on a mesoporous silica. AIChE J..

[47] Warrinnier R., Goossens T., Braun S., Gustafsson J.P., Smolders E. (2018). Modelling heterogeneous phosphate sorption kinetics on iron oxyhydroxides and soil with a continuous distribution approach. Eur. J. Soil Sci..

[48] Svitel J., Balbo A., Mariuzza R.A., Gonzales N.R., Schuck P. (2003). Combined affinity and rate constant distributions of ligand populations from experimental surface binding kinetics and equilibria. Biophys. J..

[49] Schneider C.A., Rasband W.S., Eliceiri K.W. (2012). NIH Image to ImageJ: 25 years of image analysis. Nat. Methods.

