# Supplementary material for: Extended Rate Constants Distribution (RCD) Model for Sorption in Heterogeneous Systems: 2. Importance of Diffusion Limitations for Sorption Kinetics on Cryogels in Batch
Source: Gels. 2020 May 14;6(2):15. doi: 10.3390/gels6020015 (PMC7345341; doi:10.3390/gels6020015)
Supplement: Supplementary file 1 [file gels-06-00015-s001.pdf]

Supplementary materials

Extended Rate Constants Distribution (RCD) Model for Sorption in Heterogeneous Systems: 2. Importance of Diffusion Limitations for Sorption Kinetics on Cryogels in Batch

Irina Malakhova[1], Alexey Golikov[1], Yuliya Azarova[1] and Svetlana Bratskaya[1*]

[1] Institute of Chemistry, Far Eastern Branch of Russian Academy of Sciences, 159, prosp.100-letiya Vladivostoka, Vladivostok 690022, Russia

* Correspondence: sbratska@ich.dvo.ru (S.B.), azarova87@mail.ru (Yu.A.)

Linearization of the pseudo-first-order (PFO) rate expression (1)

$$\frac{dQ_t}{dt} = k_1(Q_e - Q_t) \tag{1}$$

is given as equation (2):

$$\ln(Q_e - Q_t) = \ln Q_e - k_1 t \tag{2}$$

where Qe and Qt are the adsorbed amounts of the adsorbate at equilibrium and at time t, respectively, and k_1 is the rate constant of pseudo-first order adsorption.

The pseudo-second-order (PSO) rate expression (3), can be transformed to equation (4) and it's linear form (5):

$$\frac{dQ_t}{dt} = k_2(Q_e - Q_t)^2 \tag{3}$$

$$Qt = \frac{Q_e^2 k_2 t}{Q_e k_2 t + 1} \tag{4}$$

$$\frac{t}{Qt} = \frac{1}{k_2 Q_e^2} + \frac{1}{Q_e} t \tag{5}$$

where Q_e and Q_t are the adsorbed amounts of the adsorbate at equilibrium and at time t, respectively, and k2 is the rate constant of pseudo-second order adsorption.

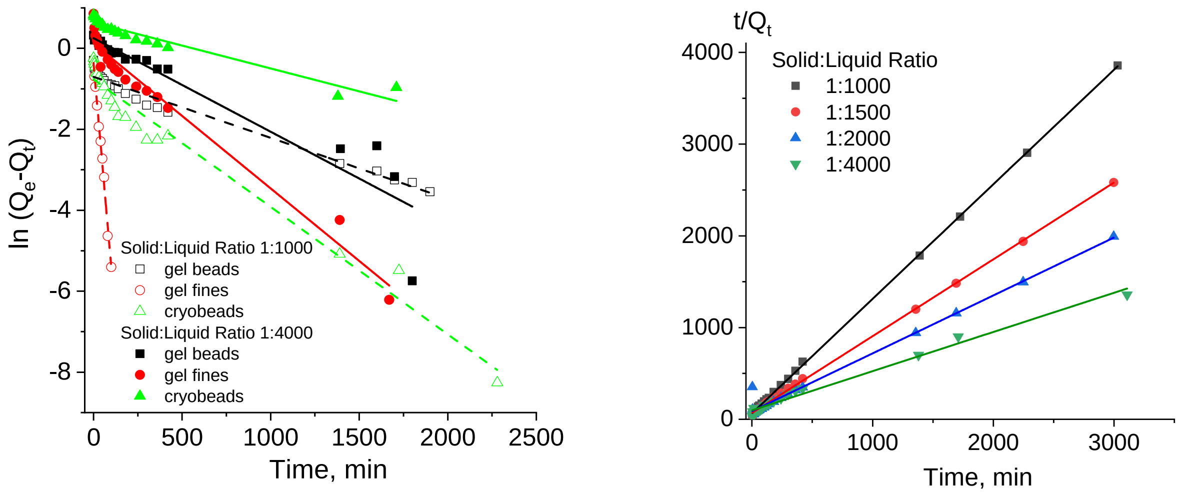

(**a**) (**b**)

Figure 1S. Linear PFO model for Cu(II) ions adsorption kinetics of PEI-gels and PEI cryobeads (a) and linear PSO model for Cu(II) ions adsorption kinetics of PEI-cryobeads (b).

Table 1S. The parameters of PFO model for kinetic curves of Cu(II) ions sorption on PEI cryogels and gels - k_1 is rate constant of pseudo-first- order adsorption, Q_e is Cu(II) adsorbed amounts at quasi-equilibrium

**Sorbent**	**Solid:liquid ratio**	**R^2**	**k_1, min^{-1}**	**Qe, mmol/g**
PEI-gel	1:1000	0,97	0,00151	0,49
PEI-gel, fines	1:1000	0,99	0,05039	0,69
PEI-cryobeads	1:1000	0,96	0,00315	0,46
PEI-gel	1:4000	0,88	0,00231	1,28
PEI-gel, fines	1:4000	0,96	0,00358	1,12
PEI-cryobeads	1:4000	0,92	0,00113	1,88

Table 2S. The parameters of PSO model for kinetic curves of Cu(II) ions sorption on PEI cryogels and gels - k_2 is rate constant of pseudo-second-order adsorption, Q_e is Cu(II) adsorbed amounts at quasi-equilibrium

**Sorbent**	**Solid:liquid ratio**	**R^2**	**k_2, g·mmol^{-1} min^{-1}**	**Qe, mmol/g**
PEI-gel, beads	1:1000	0,99	0,01194	0,76
	1:1500	0,99	0,00655	0,96
	1:2000	0,99	0,00542	1,18
	1:4000	0,97	0,00237	1,61
PEI-gel, beads (t=100 min)	1:1000	0,98	0,16579	0,38
	1:1500	0,97	0,13191	0,38
	1:2000	0,93	0,18806	0,46
	1:4000	0,47	0,02641	0,58
PEI-cryobeads	1:1000	0,99	0,02409	0,80
	1:1500	0,99	0,00996	1,20
	1:2000	0,98	0,00452	1,58
	1:4000	0,99	0,00189	2,32
PEI-cryobeads (t=100 min)	1:1000	0,98	0,05159	0,63371
	1:1500	0,94	0,02602	0,88339
	1:2000	0,98	0,01356	1,1976
	1:4000	0,94	0,03052	0,91075
PEI-gel, fines	1:1000	0,99	0,10369	0,83
	1:1500	0,99	0,06905	1,192
	1:2000	0,99	0,04156	1,59
	1:4000	0,99	0,01013	2,43
PEI-gel, fines (t=100 min)	1:1000	0,99	0,09258	0,84746
	1:1500	0,99	0,11845	1,14943
	1:2000	0,99	0,07871	1,47493
	1:4000	0,99	0,05793	1,81403

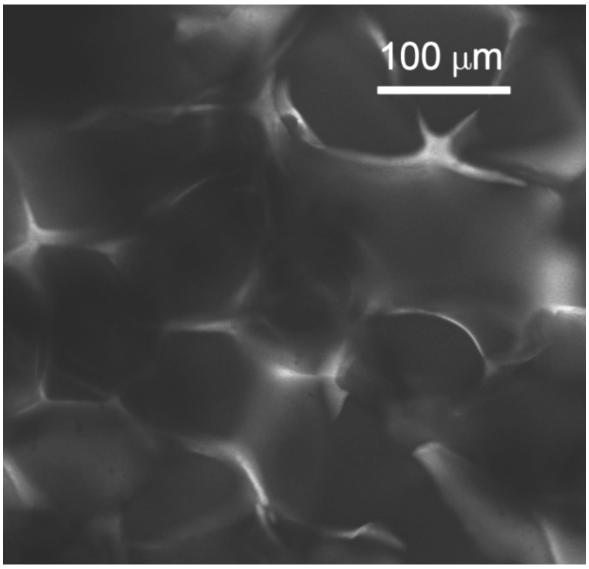

Fig. 2S. Confocal laser scanning microscopy (CLSM) images of PEI-cryogels stained with fluorescein.
